# Preliminary Clinical Experience in the Treatment of Recalcitrant Plantar Heel Pain With Stromal Vascular Fraction: Short- and Long-Term Findings From a Case Series

**DOI:** 10.7759/cureus.111420

**Published:** 2026-06-24

**Authors:** Davide Paratore

**Affiliations:** 1 Department of Orthopedics and Traumatology, Cortina Hospital, Cortina d'Ampezzo, ITA

**Keywords:** adipose-derived stromal vascular fraction, heel pain, orthobiologics, pain management, plantar fasciitis

## Abstract

This case series aims to report preliminary clinical experience in treating plantar heel pain with stromal vascular fraction (SVF) derived from adipose tissue. Four representative cases are presented. The patients were treated with a single injection of stromal vascular fraction obtained through mechanical processing of each patient’s adipose tissue. Each patient was followed up for up to 360 days after treatment. Pain was evaluated with the visual analog scale (VAS), and ultrasound (US) examination was performed at baseline and six months after treatment. A significant improvement in the VAS scale was observed in all patients within seven days. This improvement was maintained for up to 360 days. This is the first report on the treatment of plantar heel pain with SVF. These initial cases showed promising results in terms of fast and durable pain resolution. A larger clinical series is currently underway to confirm these preliminary data.

## Introduction

Plantar fasciitis and calcaneal spurs are prevalent foot conditions that substantially impact individuals' quality of life [1,2]. Plantar fasciitis is a common foot disorder caused by an inflammatory response to repetitive microtrauma [1]. Calcaneal spurs, osseous growths under the calcaneus, often coexist with plantar fasciitis [2]. The prevalence of plantar fasciitis is estimated to be around 10%, affecting various age groups and associated with factors like obesity and increased physical activity [1-3]. In 8-15% of the population, a bone spur develops in the insertional zone of the fascia (especially of the medial process) [4], where cartilaginous cells are present. Calcaneal spurs are strongly age‑related, with prevalence increasing with advancing age and large spurs being uncommon in individuals younger than 40 years [4]. A general tendency toward ossification of ligaments, such as the long plantar ligament, and tendons where they insert into bone, has been reported [4].

These conditions are characterized by intense heel pain, particularly during the initial steps of the day or after prolonged inactivity, and they can hinder mobility and significantly reduce quality of life, limiting common activities, including sports and jobs that require prolonged orthostasis [1]. Typical painful manifestations present with periods of relative remission and subsequent exacerbations. Risk factors include foot deformities such as flat foot, high arches, tight Achilles tendon and calf muscles, as the body may form a spur in response to these stressors, as well as gait abnormalities from plantar valgus or varus deformities, which place excessive stress on the heel bone [3].

Conservative management remains the first-line treatment and includes physical therapy, stretching exercises, orthotic support, and pharmacological therapies [2,5]. While many patients respond favorably, many others fail to achieve an adequate response. Importantly, conventional therapies mainly aim at symptom control rather than addressing the underlying degenerative processes, highlighting the need for regenerative strategies targeting tissue repair [2,6]. In recent years, orthobiologic approaches such as platelet-rich plasma (PRP) and cell-based therapies have been explored for their potential to promote tissue repair and modulate inflammation, including in the treatment of plantar fasciitis [5,7]. Among these, stromal vascular fraction (SVF), derived from adipose tissue, has attracted growing interest due to its heterogeneous cellular composition, including adipose-derived stromal/stem cells (ADSCs), endothelial cells, pericytes, and immune cells, which may contribute to its regenerative and immunomodulatory effects [8-10]. However, current evidence remains limited, and its clinical application in plantar fasciitis remains investigational.

From a practical and regulatory standpoint, approaches based on minimal manipulation of autologous tissues allow preparation without extensive cell processing, representing an advantageous approach. Mechanical processing systems, such as Hy-Tissue SVF, have been developed to obtain micro-fragmented connective tissue enriched with stromal vascular components, preserving elements of the native extracellular matrix while avoiding extensive processing steps [8]. These systems aim to facilitate a standardized and reproducible approach to the preparation of such biologic products in clinical settings. In our clinical practice, connective tissue micrografts-enriched SVF has been used in the management of various musculoskeletal conditions. In this case series, we present four patients with plantar fasciitis and/or calcaneal spurs refractory to conservative treatment, focusing on the safety and preliminary clinical outcomes of this approach.

## Case presentation

Four clinical cases have been selected that are representative of our larger clinical experience. The patients were diagnosed with proximal plantar fasciitis and were treated with SVF between December 2021 and July 2022. Patients below 21 years of age and pregnant women were excluded from this case series. The four included patients reported worsening of symptoms despite having previously undergone standard treatments. In detail, the patients had been symptomatic for at least six months and had been treated with nonsteroidal anti-inflammatory drugs (NSAIDs), orthotics, at least one type of treatment with physical agents (i.e., Laser, TECAR, ultrasound (US), shock waves, etc.), and at least one local infiltration with corticosteroids.

Patients’ pain was assessed at baseline and then at seven, 30, and 360 days after SVF treatment. Pain was evaluated with the 10-point visual analog scale (VAS), where the patient is asked to rate his/her level of pain, with 0 meaning ‘no pain’ and 10 meaning ‘severe pain’ [11]. The VAS is a non-proprietary psychometric methodology. The VAS does not require licensing or permission for use and has been widely adopted in clinical and research contexts since its original description in the literature. The VAS score was >8 in all cases, although each patient presented specific clinical characteristics that required individual consideration.

All patients consented to the treatment and provided informed written consent. Patients were also evaluated with US of the plantar fascia before treatment and after six months with US and standard X-ray.

Description of the technique

We treated the patients with an autologous, adipose-derived, biological product obtained with a point-of-care kit (Hy-Tissue SVF, Fidia Farmaceutici S.p.A, Abano Terme, Italy). The manufacturer had no role in the development of these case series. The system works through filtering and centrifugation, allowing to obtain microfragments of connective tissue (30-70 microns), practically without adipocytes (inert cells from the point of view of musculoskeletal regeneration). The perivascular niche is preserved, thus protecting stem cells and their function. The biological product obtained is highly concentrated, making it suitable for therapeutic applications where a small-volume injection is required.

**Figure 1:**
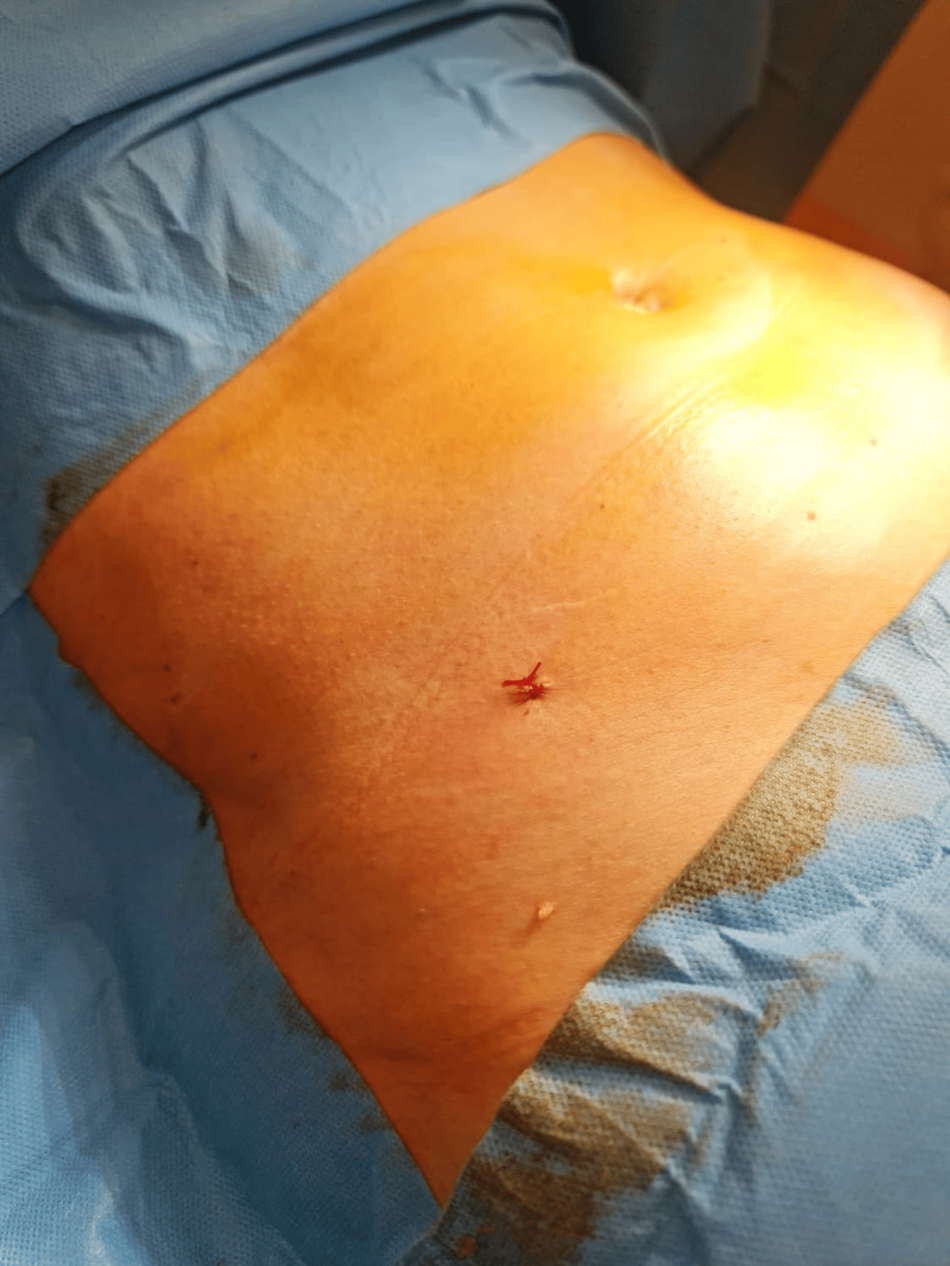
Area of fat harvesting

The patients were treated in the operating room with a one-day hospital regimen. After mild sedation of the patient with midazolam 0.03 mg/kg + propofol 0.00 mg/kg/h as a continuous intraoperative infusion, 5 cc of 2% mepivacaine was injected 1 cm proximally to the anterosuperior iliac spine on both sides. Then, 60 cc of Klein solution (epinephrine 1:1,000,000 and 0.08% mepivacaine in saline solution (0.9% NaCl)) were administered with a spinal needle into the subcutaneous tissue in the periumbilical area on both sides (Figure 1).

**Figure 2:**
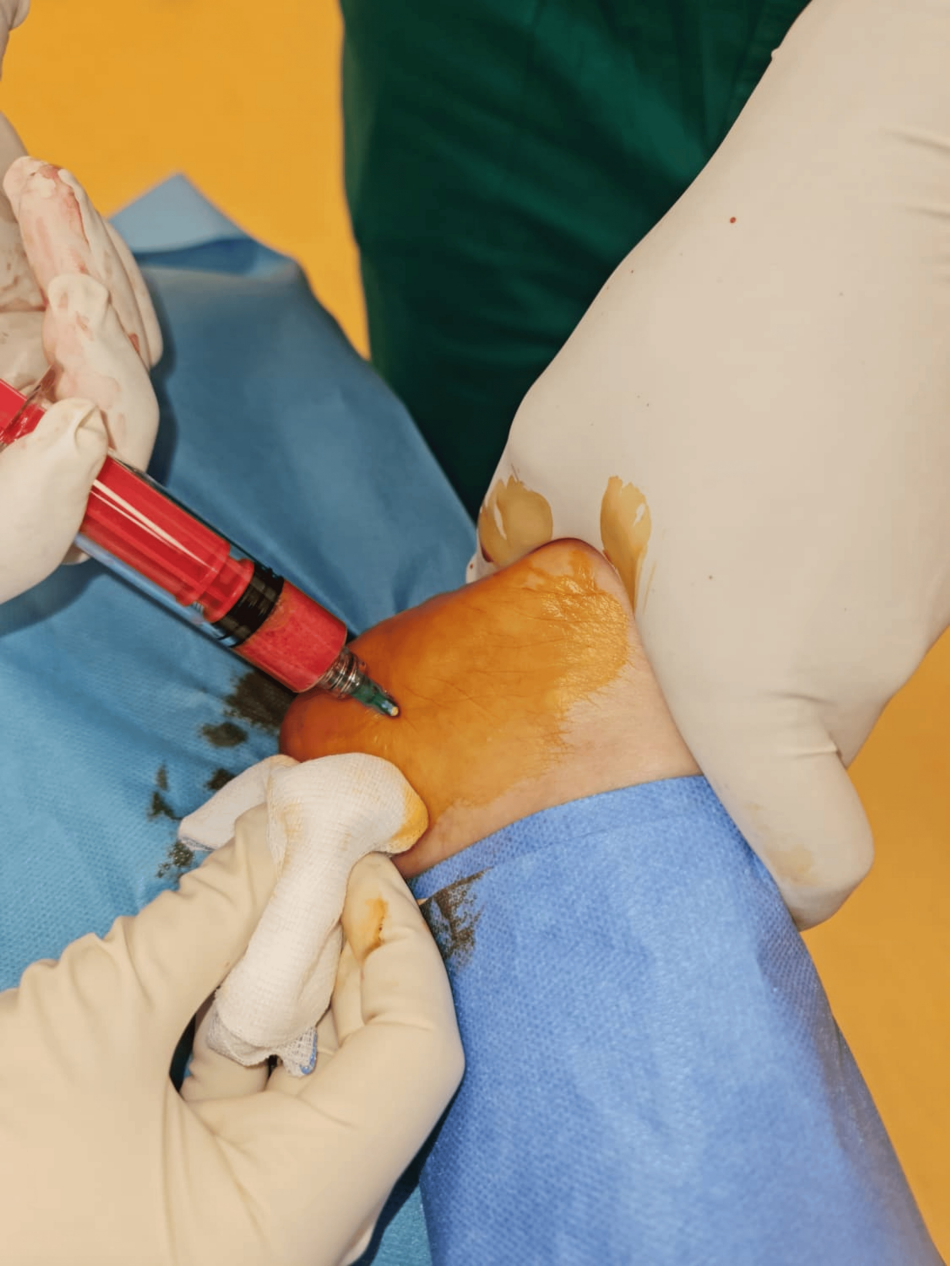
Plantar heel infiltration of SVF SVF: stromal vascular fraction

About 20 cc of subcutaneous adipose tissue was harvested through two small incisions of about 3 mm and transferred to a bag with a 120-micrometer internal filter and mechanically fragmented through a gentle massage of the contents inside the filter. The filtered tissue was collected by a syringe and centrifuged at 400 × g for 10 min. The aqueous fraction at the bottom of the syringe was further filtered through a dedicated filter and collected. In total, about 10 cc of preparation were obtained and injected medially at the proximal attachment of the plantar fascia (Figure 2).

In the meantime, the small abdominal portals served for sampling were sutured, medicated, and a hypoallergenic transparent film was applied to the abdominal region where the fat was taken to avoid abundant bruising.

Case 1

A 62-year-old male with a history of insulin-dependent diabetes mellitus presented with an eight-month history of progressive right heel pain, consistent with plantar fasciitis associated with a calcaneal spur (revealed by X-ray). A history of chronic low back pain was reported, but it was not contributory to the presenting complaint. The onset of symptoms was insidious, with gradual worsening over time. Pain was predominantly localized to the plantar aspect of the heel and was exacerbated by weight-bearing activities, including standing and ambulation.

Initial management by the primary care physician consisted of oral NSAIDs (ibuprofen 600 mg twice daily for one week), resulting in only partial and temporary relief, with recurrence of symptoms upon discontinuation. The patient also tried conservative biomechanical interventions, including insoles of varying densities and configurations, as well as silicone heel cups, without any sustained benefit.

Two months after the symptom onset, the patient underwent local depot corticosteroid injections. These provided transient symptomatic relief lasting approximately seven days; however, symptoms recurred following the second injection. At approximately six months from symptom onset, the patient was referred for specialist evaluation due to persistent symptoms. At that time, the condition had significantly impacted functional status, limiting occupational activities to a few hours per day and leading to cessation of long-distance walking. The patient reported pain during standing. Physical examination revealed marked tenderness over the medial plantar of the heel.

The preoperative VAS score for pain was 10/10. The patient subsequently underwent treatment with SVF using the Hy-Tissue SVF system. At seven days post-procedure, a marked clinical improvement was observed, with a reduction in VAS score to 2/10. The improvement was associated with restoration of functional capacity, including the ability to resume prolonged walking (up to approximately 13 km per day).

Case 2

A 52-year-old male with a sedentary occupation but an active lifestyle (mountain running) presented with left plantar heel pain consistent with plantar fasciitis associated with a calcaneal spur. Symptom onset followed a prolonged walk on a hard surface, after which pain persisted during both standing and ambulation. Initial management included oral NSAIDs (ibuprofen 400 mg twice daily), without clinical benefit. The patient subsequently received intramuscular ketorolac (two administrations daily for three days), which provided only transient pain relief lasting approximately two hours after each injection.

Additional conservative therapies included 10 sessions of US therapy and six sessions of TECAR therapy, with no improvement. The patient also used silicone heel cups without benefit. Extracorporeal shockwave therapy was initiated (four sessions performed; treatment discontinued before completion of the prescribed six sessions) without meaningful symptom relief. On clinical examination, marked tenderness was elicited at the inferomedial aspect of the calcaneus, corresponding to the plantar fascia insertion. Associated findings included contralateral hallux valgus and a pronation syndrome, which was less pronounced on the affected side. Pain persisted and worsened during weight-bearing activities.

At six months from symptom onset, the patient underwent treatment with SVF using the Hy-Tissue system. The preoperative VAS score was 9/10. At seven days post-treatment, complete resolution of pain was reported (VAS 0/10), allowing immediate return to work. Approximately 30 days after SVF treatment, the patient underwent corrective surgery for contralateral hallux valgus and pronation syndrome. At approximately 80 days post-SVF treatment, the patient gradually resumed athletic activity. No recurrence of symptoms was reported at one-year follow-up.

Case 3

A 45-year-old female bartender presented with a history of left plantar heel pain, initially reported in 2018, localized predominantly to the medial plantar aspect of the heel and reaching a VAS score of 9/10. Initial management included three local corticosteroid injections administered at weekly intervals, resulting in only transient symptom relief. Additional therapies included six sessions of extracorporeal shockwave therapy, massage therapy, and both active and passive kinesiotherapy, all without sustained clinical benefit.

Approximately three months after symptom onset, pain extended to involve the entire plantar fascia during weight-bearing. In 2019, the patient underwent percutaneous excision of the calcaneal spur combined with medial plantar fasciotomy. Initial improvement was noted after one week; however, weight-bearing remained limited. After three months of rehabilitation, residual pain persisted (VAS 4/10), along with metatarsal pain (involving the second and third toes). The metatarsal symptoms were subsequently managed with a percutaneous osteotomy, followed by a three-month rehabilitation period, resulting in complete resolution of pain (VAS 0/10).

In 2021, the patient developed similar symptoms in the contralateral foot. Given the previous prolonged recovery and occupational constraints, the patient declined repeat surgical management. Due to progressive symptoms involving the entire plantar fascia, treatment with SVF (Hy-Tissue system) was proposed and performed approximately one year after symptom onset. At seven days post-procedure, the patient reported complete pain resolution (VAS 0/10) and resumed occupational activities without limitation. No recurrence of symptoms was reported at the one-year follow-up.

Case 4

A 70-year-old female working in the hospitality sector presented with progressively worsening right plantar heel pain localized to the inferomedial region of the right heel. Initial conservative management with silicone heel cups resulted in partial symptom relief (VAS reduction from 8/10 to 6/10); however, pain persisted after approximately one month. Pharmacological treatments included COX-2 inhibitors, topical and oral NSAIDs, and short courses of oral corticosteroids (seven-day cycles), without sustained improvement.

The patient also underwent 10 sessions of physiotherapy and TECAR therapy, with only mild and temporary benefit. At approximately two years from symptom onset, the patient underwent a local depot corticosteroid injection, resulting in partial and short-lived symptom relief (VAS reduction from 9/10 to 6/10, lasting approximately five days). The injection was associated with adverse effects, including facial flushing, transient hypertension (up to 170/95 mmHg), and hot flashes lasting approximately 48 hours.

Given persistent symptoms, the patient underwent treatment with SVF using the Hy-Tissue system. At three days post-procedure, the patient returned to normal daily and social activities. At the seven-day follow-up, the VAS score was 0/10. The patient regained the ability to wear closed shoes, which had previously been intolerable. Clinical improvement was maintained at the one-year follow-up, with no recurrence of symptoms.

Summary of case results

Significant improvement in the VAS scale was achieved in all patients within seven days. No use of any type of anti-inflammatory drug or any other additional treatment was necessary. Functionally, all patients resumed their normal activities within seven days without any kind of limitations. The result was obtained with a single SVF treatment session. No adverse effects have been reported, confirming the safety profile of SVF treatment. The main characteristics and observations of the four cases are summarized in Table 1.

**Table 1:** Patient characteristics and results of SVF treatment evaluated by VAS* ^*^[11] SVF: stromal vascular fraction; VAS: visual analog scale; FU: follow-up; NSAIDs: nonsteroidal anti-inflammatory drugs; NA: not applicable

Case	Patient profile		Clinical signs	Duration of the disease	Diagnosis	Previous treatments	Comorbidities	Initial VAS	Post-Treatment VAS (7 days)	VAS at 30 days	Improvement at 1-year FU
1	62-year-old male		Heel pain, lumbago	8 months	Calcaneal spur/plantar fasciitis	NSAIDs, corticosteroid injections, and footwear adjustments	Diabetes, hypertension	10/10	2/10	2/10	Maintained VAS: 1
2	52-year-old male		Heel pain	6 months	Calcaneal spur/plantar fasciitis	NSAIDs, ultrasound therapy (5 sessions)	Contralateral hallux valgus	9/10	1/10	0/10	Maintained VAS: 0
3	45-year-old female		Heel pain	1 year	Calcaneal spur/plantar fasciitis	N/A	Previously operated contralaterally for the same problem with percutaneous exostosectomy (recovery after 6 months)	10/10	1/10	0/10	Maintained VAS: 0
4	70-year-old female		Heel pain	2 years	Calcaneal spur/plantar fasciitis	NSAIDs, fisiokinesiterapia, tacareterapia, corticosteroid injections	N/A	9/10	0/10	0/10	Maintained VAS: 0

**Figure 3:**
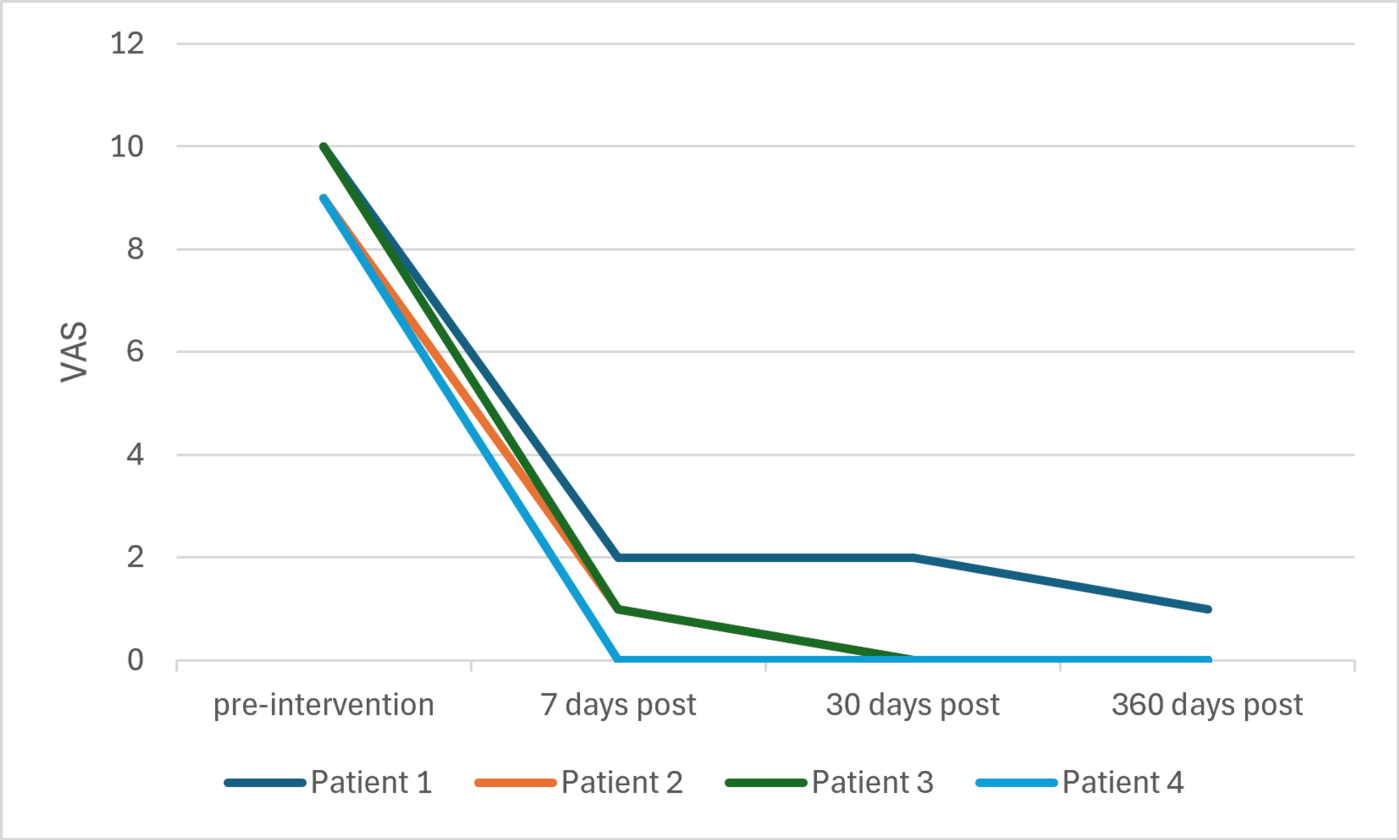
Results over time from baseline to 1-year post-SVF treatment SVF: stromal vascular fraction; VAS: visual analog scale

The VAS trend over time for each patient is depicted in Figure 3.

**Figure 4:**
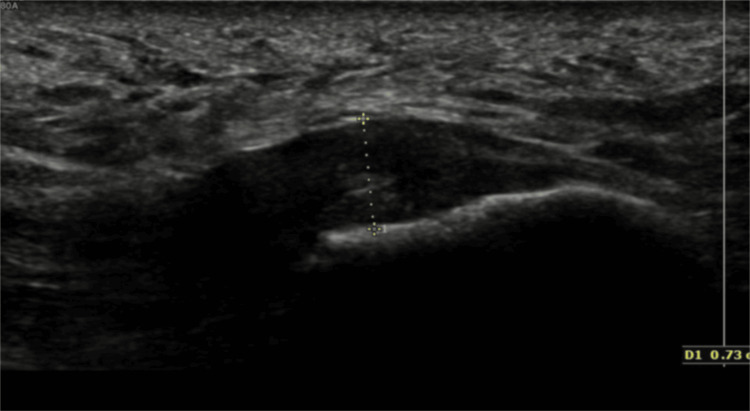
Representative pre-treatment US image showing thickening of the plantar fascia (baseline) - case 3 US: ultrasound

On radiographic examination, all patients had plantar heel spurs confirmed by US. Patients undergoing US also presented thickening of the plantar fascia at the proximal insertion and areas of hypoechogenicity of the structure (representative image is presented in Figure 4).

**Figure 5:**
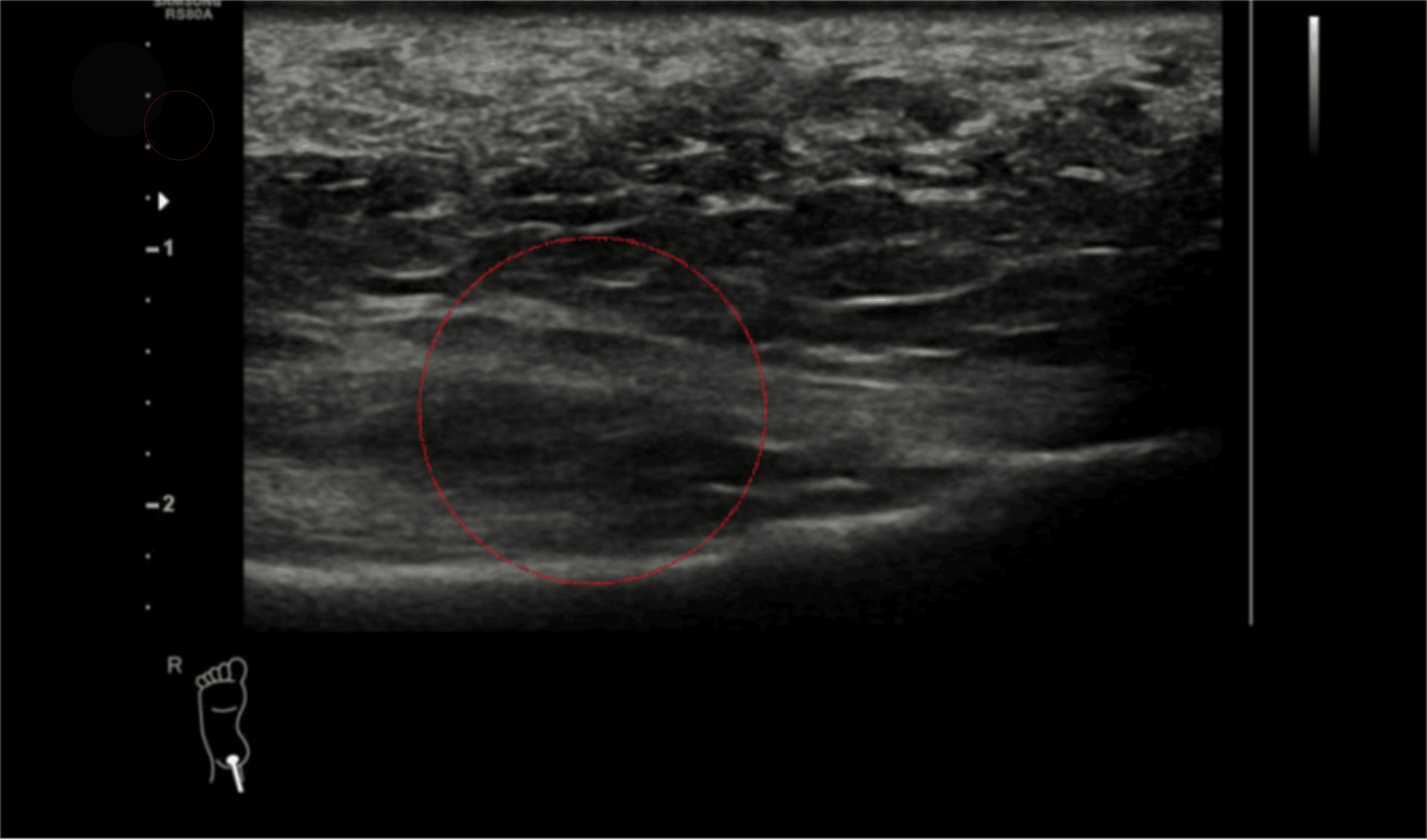
Representative US image showing reduction in the thickness of the fascia at six months after treatment with SVF - case 3 US: ultrasound; SVF: stromal vascular fraction

All patients underwent an ultrasound examination approximately six months after treatment, which showed a modest but clinically significant reduction in the thickness of the fascia (approximately 2 mm) and an almost complete disappearance of the hypoechogenic areas (representative image is provided in Figure 5).

In one case (case 3), incidentally, the patient underwent radiographic examination after two years: the spur, originally present, was reduced by approximately 4 mm.

## Discussion

The main objective of this initial clinical experience was to obtain significant regression of plantar heel pain symptoms as quickly as possible, excluding prolonged periods of convalescence and, therefore, inactivity of patients. The concept of using SVF was inspired by previous studies that have addressed Achilles tendon pathology [12,13], which is closely related to plantar fasciitis, as it shares the same functional system. Nevertheless, this appears to be the first clinical experience involving treatment with SVF in this widespread condition. SVF is obtained from autologous fat tissue via liposuction, which is then processed to separate the cellular fraction. This processing can be performed by enzymatic or mechanical means. Of note, mechanical methods result in lower nucleated cell yields, but they are classified by the U.S. Food and Drug Administration (FDA) and European Medicines Agency (EMA) as “minimal manipulation,” requiring less regulatory approval, which potentially simplifies clinical use [14]. Moreover, mechanical processing may be preferable to enzymatic isolation because no exogenous enzymes are used, and the native three-dimensional (3D) anatomical structure is preserved [15].

Considering the results reported in this case series and other observations from routine clinical practice at our institution, it can be stated that the structure of the plantar fascia, given its purely connective nature, is highly responsive to treatment with SVF. In patients affected by plantar heel pain, in fact, the fascia is subjected to supraphysiological stress leading to microtears, which is thought to induce an acute inflammatory response [16]. The anti-inflammatory action of SVF is probably related to the paracrine effect of some specific cells, such as M2 macrophages, which are present within this heterogeneous cell population [17]. On the other hand, our preliminary radiological observations highlight an improvement in the examined area, which seems to suggest that the role of SVF derived from adipose tissue is not only related to a powerful anti-inflammatory action, but may also be involved in initiating a reparative and regenerative process.

Interestingly, recent results in a preclinical model of partial-thickness Achilles tendon defect have suggested the healing potential of uncultured adipose-derived cells in tendon tears [18]. In fact, the authors reported that a single injection of uncultured, unmodified autologous adipose-derived regenerative cells into the lesion was able to promote the formation of biomechanically functional, histologically organized new connective tissue with significantly better structural integration and less scar formation than after sham treatment. Additionally, other studies suggest regenerative properties of SVF injections. For instance, in the treatment of osteoarthrosis, SVF injections have not only been shown to reduce pain and improve joint function, but imaging evidence has also suggested a cartilage-healing effect [19].

In line with other studies on SVF use, no adverse effects have been reported in the treated cases, confirming the safety of SVF injections for musculoskeletal applications [19,20]. To date, in our hospital, approximately 30 additional patients have been successfully treated with SVF injections for plantar heel pain related to heel spurs and/or plantar fasciitis, and long-term assessment is ongoing to further confirm and complete the clinical observations described in this case series.

## Conclusions

The treatment of lower plantar heel pain with a single SVF injection in patients who had previously been treated unsuccessfully with standard non-surgical therapies has shown encouraging outcomes, with both immediate and sustained reductions in pain. Preliminary ultrasound findings also indicate possible improvements in plantar fascia structure following treatment. The rapid resolution of the symptoms, the prolonged duration of the effects, the extreme practicality, and the minimal surgical impact make SVF a very promising solution for patients presenting with recalcitrant plantar fasciitis and calcaneal spurs. Overall, these preliminary findings suggest that SVF may represent a safe and potentially effective therapeutic option for the treatment of plantar fasciitis. Nevertheless, given the limited size of this case series, larger studies with extended follow-up are needed to confirm these preliminary results, which may represent a starting point for further investigation into the clinical application of stromal vascular fraction obtained from mechanically processed lipoaspirate as a potential option for the treatment of plantar heel pain.
